# Metabolic and proteomic analyses of product selectivity and redox regulation in *Clostridium pasteurianum* grown on glycerol under varied iron availability

**DOI:** 10.1186/s12934-017-0678-9

**Published:** 2017-04-19

**Authors:** Christin Groeger, Wei Wang, Wael Sabra, Tyll Utesch, An-Ping Zeng

**Affiliations:** Institute of Bioprocess and Biosystems Engineering, Hamburg University of Technology, Denickestr.15, 21073 Hamburg, Germany

**Keywords:** *n*-Butanol, 1,3-Propanediol, *C. pasteurianum*, Proteomics, Product selectivity, Metabolic analysis

## Abstract

**Background:**

*Clostridium pasteurianum* as an emerging new microbial cell factory can produce both *n*-butanol (BuOH) and 1,3-propanediol (1,3-PDO), and the pattern of product formation changes significantly with the composition of the culture medium. Among others iron content in the medium was shown to strongly affect the products selectivity. However, the mechanism behind this metabolic regulation is still unclear. For a better understanding of such metabolic regulation and for process optimization, we carried out fermentation experiments under either iron excess or iron limitation conditions, and performed metabolic, stoichiometric and proteomic analyses.

**Results:**

1,3-PDO is most effectively produced under iron limited condition (Fe−), whereas 1,3-PDO and BuOH were both produced under iron rich condition (Fe+). With increased iron availability the BuOH/1,3-PDO ratio increased significantly from 0.27 mol/mol (at Fe−) to 1.4 mol/mol (at Fe+). Additionally, hydrogen production was enhanced significantly under Fe+ condition. Proteomic analysis revealed differentiated expression of many proteins including several ones of the central carbon metabolic pathway. Among others, pyruvate: ferredoxin oxidoreductase, hydrogenases, and several electron transfer flavoproteins was found to be strongly up-regulated under Fe+ condition, pointing to their strong involvement in the regeneration of the oxidized form of ferredoxin, and consequently their influences on the product selectivity in *C. pasteurianum*. Of particular significance is the finding that H_2_ formation in *C. pasteurianum* is coupled to the ferredoxin-dependent butyryl-CoA dehydrogenase catalyzed reaction, which significantly affects the redox balance and thus the product selectivity.

**Conclusions:**

The metabolic, stoichiometric and proteomic results clearly show the key roles of hydrogenases and ferredoxins dependent reactions in determining the internal redox balance and hence product selectivity. Not only the NADH pool but also the regulation of the ferredoxin pool could explain such product variation under different iron conditions.

## Background

*Clostridium pasteurianum* is an emerging and promising microbial cell factory for the production of chemicals and fuels because of some unique features, e.g. utilization of a wide range of substrates [1–3], production of a wide spectrum of products [4, 5] and robust growth in simple media even under unsterile conditions [6]. Recently, *C. pasteurianum* was shown to accept electrons from the cathode by direct electron transfer [7], which make it an attractive candidate for new bioelectrical systems. Therefore, *C. pasteurianum* has received considerable interests for the production of chemicals and fuels such as 1,3-propanediol (1,3-PDO) and *n*-butanol (BuOH), which represents attractive bioprocesses for the use of renewable resources, like biodiesel-derived glycerol or glucose from biomass hydrolysates [8–14]. In such bioprocesses, several other fermentation products like gases (carbon dioxide and hydrogen), ethanol as well as acetic, butyric and lactic acid are produced [2, 4, 15], in addition to 1,3-PDO and BuOH. Even though the formation of organic acids is inevitable for the maintenance of the intracellular redox balance, it represents a loss of carbons at the expense of the target products. Moreover, the resulting product distribution, especially the selectivity of either 1,3-PDO or BuOH, is mainly influenced by the cultivation conditions and/or media supplements. For instances, several studies analyzed the effect of pH [16], inoculum conditions [17], supplementations of yeast extracts and ammonia [18], or acetic and butyric acid [2, 19], or phosphate and iron [18, 20]. Among others, iron seems to have extensive effects, since its absence lead to a strongly reduced BuOH formation [16, 20]. In real fermentation processes, especially under conditions more relevant to industrial applications, with raw substrates and high concentrations of products, the product selectivity and yield often strongly fluctuate and are hardly reproducible. The underlying mechanism(s) of selectivity and regulation of intracellular metabolic pathways are still unclear, even though a combined effect of many iron-related enzymes has been assumed [4]. Indeed, several iron containing enzymes are involved in *clostridia* metabolism, e.g. nitrogenases, ferredoxin coupled enzymes, and alcohol dehydrogenases. These enzymes play key roles in the maintenance of intracellular redox balance and a limited functionality of them, e.g. due to iron limitation, will be reflected by a metabolic shift and thus change of product selectivity.

In this work, the variations of product selectivity and the underlying mechanisms of pathway regulation in *C. pasteurianum* DSMZ 525 grown on glycerol under varied iron availability are studied with an integrated systems biology approach, particularly with stoichiometric, kinetic and proteomic analyses.

## Methods

### Microorganism, medium and cultivation

*Clostridium pasteurianum* DSMZ 525 was routinely maintained as cryoculture at −80 °C in Reinforced Clostridial Medium (RCM, Oxoid Deutschland GmbH) containing 20% (v/v) glycerin. The cryoculture was used for the pre-culture carried out in anaerobic bottles with RCM medium at 35 °C and pH 7 without shaking. The RCM contained 1 mg/L resazurin (7-hydroxy-10-oxidophenoxazin-10-ium-3-one) as a redox indicator for anaerobiosis and 2 g/L CaCO_3_ as pH-buffering agent. After 24 h this pre-culture was used as inoculum for bioreactor fermentation. The bioreactor medium contained the following ingredients in 1 L of distilled water (modified from [4]): glycerol, 80 g; yeast extract, 1 g; K_2_HPO_4_, 0.5 g; KH_2_PO_4_, 0.5 g; MgSO_4_·7H_2_0, 0.2 g; (NH_4_)_2_SO_4_, 5 g; CaCl_2_ 2H_2_O, 0.02 g; cysteine-HCl, 0.5 g; resarzurin, 0.005 g; trace element solution SL-7 (DSMZ), 2 mL. Iron concentrations were varied in the bioreactor medium as follows: Iron excess (Fe+) condition means the addition of 10 mg/L FeSO_4_·7H_2_O (2 mg Fe^2+^/L) into the medium and iron limitation (Fe−) condition means no iron addition. Iron originally present in the pre-culture (0.07 mg Fe^2+^/L) and those present in the yeast extract (up to 0.05 mg Fe^2+^/L) were the sole iron sources in the Fe− cultivations. Cultivations were run at 35 °C, pH 6 and 500 rpm agitation in a stirred tank bioreactor (Bioengineering) with a working volume of 1.2 L. During the fermentation pH was maintained at 6 with 5 M KOH. To achieve anaerobic condition prior to the inoculation, the autoclaved medium was sparged with sterile O_2_-free N_2_. The experiments were performed in duplicates. Total volume of the effluent fermentation gas was determined with a Milli-Gascounter (Dr.-Ing. Ritter Apparatebau GmbH & Co. KG), and its composition was measured with the mass spectrometer OmniStar 300 (Balzer Instruments/Pfeiffer Vacuum GmbH). The MS took samples in an interval of 0.5 mL/min for the concentration analysis of H_2_, CO_2_, O_2_, N_2_ and Ar.

### Analytical methods and calculations

The optical density of cell suspension was measured turbidometrically at 600 nm and correlated with cell dry weight: biomass BM (g/L) = OD_600_ × 0.336. The specific growth rate µ (h^−1^) was determined from biomass data (smoothed using the software Origin 8.5.1 G SR1, OriginLab Corporation, Northampton, USA) according to Eq. 1, where x_1_ and x_2_ are the concentrations of biomass (g/L) at the times t_1_ and t_2_, respectively. The substrate and product titers in the supernatant were analyzed via HPLC equipped with a refractive index detector and an ultraviolet detector. HPLC was performed on an Aminex HPX-87H column (300 × 7.8 mm) at 60 °C, with 0.005 M H_2_SO_4_ as mobile phase at a flow rate of 0.6 mL/min.

For the measurement of 3-HPA, the method described by Oehmke and Zeng [21] was used, in which 3-HPA is converted into acrolein and the concentration of acrolein is determined spectrometrically by external calibration. Briefly, 100 µL of cell free culture supernatant were mixed with 200 µL of HCl (37%) and 50 µL of tryptophan solution in a cooled 96 well plate. The tryptophan solution consisted of 10 mM DL-tryptophan, 0.05 M HCl and 24 mM toluene. After 40 min incubation at 37 °C, the absorbance of the mixtures were determined with a Multiskan^®^ Spectrum plate reader (Thermo Fisher Scientific) at 560 nm.

The yield coefficient (Y) for either product or substrate (i) was calculated according to Eq. 2. Based on the stoichiometric equations for glycerol utilization in *C. pasteurianum* [11], carbon and redox recovery were calculated according to Eqs. 3 and 4, respectively. Here C [−] is the number of carbon atoms in the products and substrate, c is the concentration of products in (mmol/L) and biomass (BM) in (g/L).

1 $$\upmu = \frac{{\ln {\text{x}}_{2} - \ln {\text{x}}_{1} }}{{{\text{t}}_{2} - {\text{t}}_{1} }}$$

2 $${\text{Y}}_{{{\text{i}}/{\text{X}}}} = \frac{{{\text{i}}_{2} - {\text{i}}_{1} }}{{{\text{x}}_{2} - {\text{x}}_{1} }}$$

3 $${\text{C}}_{\text{recovery}}\, [{\text{\%}}] = \frac{{\mathop \sum \nolimits {\text{C}}_{\text{products }} }}{{\mathop \sum \nolimits {\text{C}}_{\text{substrate}} }}$$

4 $${\text{NADH}}_{\text{recovery}}\, [{\text{\%}}] = \frac{{{\text{c}}_{{1,3 - {\text{PDO}}}} }}{{2 {\text{c}}_{\text{acetate}} + 2 {\text{c}}_{\text{butyrate}} + {\text{c}}_{\text{lactate}} + 13.2 {\text{c}}_{\text{BM}} }}$$

### Comparative proteomic analysis

Samples for proteomics were taken in the exponential growth phase and stationary phase during parallel fermentations of *C. pasteurianum* DSMZ 525 under iron excess and iron limited conditions. The detailed methodical procedure for comparative proteomic analysis was previously described by Sabra et al. [11].

## Results and discussion

### Effects of iron availability on the growth and product formation of *C. pasteurianum*

**Fig. 1:**
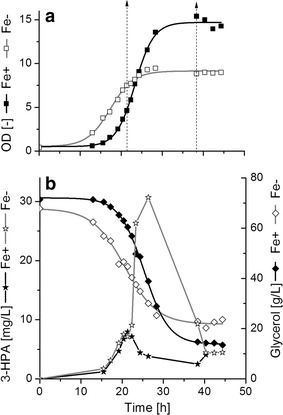
**a** Cell growth behavior and **b** 3-HPA formation and glycerol consumption in cultivation of *C. pasteurianum* DSMZ 525 under iron excess (Fe+) and iron limitation (Fe−) conditions. *Arrows* indicate time points of sampling for proteomic analysis

Different concentrations of iron have been reported for the optimization of butanol or 1,3-PDO formation using *C. pasteurianum* [2, 18, 20]. Using a fractional factorial experimental design, Moon et al. used 60 mg/L FeSO_4_·7H_2_O for optimum butanol formation in *C. pasteurianum* in serum anaerobic bottle experiments, while no iron sulphate was supplemented for a better 1,3-PDO production [18]. In controlled bioreactor, we have found that 10 mg/L FeSO_4_·7H_2_O is enough to support a similar butanol productivities (0.9 g/L×h) and an almost doubled butanol concentration (21 g/L butanol) by the same strain [2]. Therefore, in the current investigation, 0 and 10 mg/L FeSO_4_·7H_2_O were chosen, respectively, to describe the growth and product formation under iron limited and iron excess conditions in our glycerol fermentation. The same pre-culture was used to inoculate two bioreactors containing the growth medium supplemented either with or without 10 mg/L FeSO_4_·7H_2_O (hereinafter termed as iron excess (Fe+) condition or iron limitation (Fe−) condition, respectively). One of the main differences observed was the relatively shorter lag phase under Fe− condition, which was accompanied by an early growth cessation (Fig. 1a). With excess iron in the medium, a higher biomass production with a maximum concentration of 5.1 ± 0.09 g/L and µ_max_ of 0.31 ± 0.01 h^−1^ were reached, whereas under iron limitation condition only a biomass concentration of 3.2 ± 0.01 g/L and a µ_max_ of 0.23 ± 0.01 h^−1^ could be achieved (Table 1). The cessation of growth under iron limitation was obviously not due to butanol toxicity, as the highest titer of BuOH reached did not exceed 3.7 g/L (Table 1), which was lower than the toxic concentration level of BuOH for *C. pasteurianum* (>5 g/L [11]). Depletion of the intracellular iron pool and/or the accumulation of 3-hydroxypropionaldehyde (3-HPA), a very toxic intermediate in the formation of 1,3-PDO [22, 23], may cause the relatively earlier growth cessation under Fe− condition. As shown in Fig. 1b, under Fe+ condition the 3-HPA concentration did not exceed 8 mg/L, whereas under Fe− condition up to 30 mg/L 3-HPA were produced. In this time range of relatively high concentrations of 3-HPA a growth cessation was observed. Indeed, it has been reported that the growth of vegetative cells of *C. tyrobutyricum* was completely inhibited at 38 mg/L externally added 3-HPA [24].

**Table 1:** Product formation during the growth of *C. pasteurianum* DSMZ 525 under iron excess (Fe+) and iron limitation (Fe−) conditions. Fermentations were performed in duplicates

	Biomass		1,3-PDO		BuOH		EtOH		Acetate		Butyrate		Lactate		Formate	
	Fe+	Fe−	Fe+	Fe−	Fe+	Fe−	Fe+	Fe−	Fe+	Fe−	Fe+	Fe−	Fe+	Fe−	Fe+	Fe−
Final titer (g/L)	5.1 ± 0.09	3.2 ± 0.01	9.4 ± 1.44	16.6 ± 2.26	12.3 ± 0.06	4.4 ± 0.70	1.0 ± 0.12	0.9 ± 0.60	0.8 ± 0.01	1.5 ± 0.02	1.7 ± 0.45	2.8 ± 0.13	0.1 ± 0.00	7.5 ± 2.58	0.5 ± 0.07	1.1 ± 0.11
Production rate (g/L*h) at exponent-ial growth phase	0.39 ± 0.00	0.16 ± 0.03	0.66 ± 0.17	0.74 ± 0.02	0.53 ± 0.11	0.16 ± 0.04	0.06 ± 0.02	0.03 ± 0.003	0.04 ± 0.004	0.07 ± 0.03	0.14 ± 0.02	0.13 ± 0.01	0.007 ± 0.003	0.28 ± 0.02	0.03 ± 0.01	0.04 ± 0.02
	Y S/X		Y P/X													
Y _i/X_ (g/g BM)	9.6 ± 0.3	13.9 ± 1.2	1.16 ± 0.09	4.40 ± 0.52	1.88 ± 0.25	0.97 ± 0.13	0.15 ± 0.06	0.17 ± 0.13	0.10 ± 0.01	0.39 ± 0.09	0.39 ± 0.08	0.80 ± 0.17	0.02 ± 0.00	1.64 ± 0.37	0.08 ± 0.02	0.25 ± 0.05

**Fig. 2:**
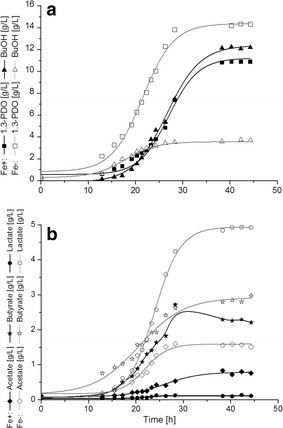
1,3-PDO and BuOH formation (**a**) and acid formation (**b**) in *C. pasteurianum* DSMZ 525 during glycerol fermentation under iron excess (Fe+) and iron limitation (Fe−) conditions

Figure 2 shows the formation of fermentation products in *C. pasteurianum DSMZ 525* under iron excess and iron limitation conditions. With higher iron concentration 12 g/L of BuOH and 11 g/L of 1,3-PDO were produced. In comparison, at iron limitation significantly less BuOH, i.e. 3.7 g/L, was formed, accompanied with the formation of 14.5 g/L of 1,3-PDO. In fact, the molar ratio of BuOH to 1,3-PDO decreased from 1.34 mol/mol under Fe+ condition to 0.27 mol/mol under Fe− condition. The yield of 1,3-PDO per biomass increased nearly 4 times from 1.16 ± 0.09 g/g at Fe+ to 4.4 ± 0.52 g/g at Fe−, whereas the specific yield of BuOH was halved from 1.88 ± 0.25 g/g at Fe+ to 0.97 ± 0.13 g/g at Fe−. Next to this, the acid formation changed remarkably, especially the lactate production, which was shown to increase significantly in the Fe− culture (Table 1). The specific lactate yield increased significantly from 0.02 ± 0.0 g/g in the Fe+ culture to 1.6 ± 0.4 g/g in the Fe− culture. Also acetate and butyrate yield increased under Fe− condition, but to less extent than that of lactate (Table 1). The reason(s) for these dramatic changes of metabolism are not clear yet, but of fundamental importance for the development of *C. pasteurianum* as an emerging microbial cell factory for the production of chemicals and fuels. Therefore in the following redox regulation and comparative proteomic analysis were performed.

### Redox regulation and H_2_ production in *C. pasteurianum* DSMZ 525

For the growth and metabolism of *C. pasteurianum*, particularly when growing on a more reduced substrate like glycerol, the maintenance of intracellular redox balance is crucial. The shift of metabolism under conditions of iron excess and limitation shown above is postulated to be strongly related to the redox regulation which is addressed below first from a stoichiometric point of view.

To check the stoichiometry and consistency of the fermentation data, fermentation balance analysis was first done. A very good consistency in carbon recovery was observed for the fermentations. The carbon recovered as fermentation products represented approximately 98% of the carbon source consumed. On the other hand, the calculated recovery of the reducing equivalents according to Eq. 4 reached 91% at Fe+ and 94% at Fe−, indicating a lower consistency in reducing equivalent recovery according to the assumed pathways of redox regulation. *C. pasteurianum* contains ferredoxin-dependent hydrogenases, which catalyze the re-oxidation of reduced ferredoxin with the formation of H_2_. Reduced ferredoxins are generally formed in the enzymatic step of forming acetyl-CoA from pyruvate catalyzed by pyruvate: ferredoxin oxidoreductase (PFOR). Hence, under the assumption that the formation of one mole acetyl-CoA from pyruvate is accompanied with the formation of one mole H_2_, the theoretical H_2_ production would be calculated according to Eq. 5, where q is the formation rate of each compound (mmol/g×h):

5 $${\text{q}}_{{{\text{H}}2}} = {\text{q}}_{\text{ethanol}} + {\text{q}}_{\text{acetate}} + 2 {\text{q}}_{\text{butyrate}} + 2 {\text{q}}_{\text{butanol}}$$

**Fig. 3:**
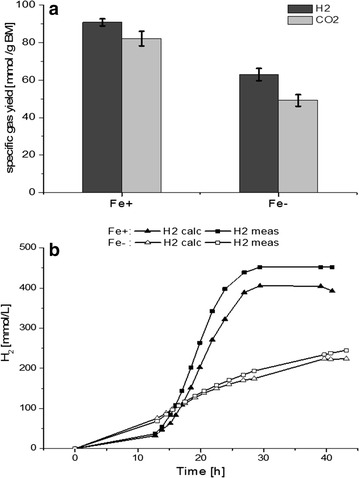
**a** Measured cumulative hydrogen and carbon dioxide production and **b** calculated (calc) vs. measured (meas) hydrogen production under iron excess (Fe+) and iron limitation (Fe−) conditions in *C. pasteurianum* DSMZ 525 cultures

In repeated fermentations to those shown in Fig. 1 under similar conditions we measured the evolution of CO_2_ and H_2_ in effluent gas. The results are given in Fig. 3a. Under Fe+ conditions a cumulative amount of 452 mmol/L H_2_ and 399 mmol/L CO_2_ were produced, compared to 245 mmol/L H_2_ and 177 mmol/L CO_2_ produced under Fe− condition. Referred to the biomass formed, H_2_ and CO_2_ production increased significantly from 63 (±3.3) and 49 (±3.1) mmol/g _biomass_ at Fe− condition to 91 (±1.9) and 82 (±3.9) mmol/g _biomass_ at Fe+ condition, respectively. Interestingly, the theoretically calculated H_2_ production values were lower than the measured ones (Fig. 3b), particularly under Fe+ conditions. Obviously, the re-oxidation of reduced ferredoxin generated in the enzymatic step catalyzed by PFOR was not the only source of hydrogen formation. Similar behavior was also noticed previously in cultures of *C. butyricum* or *Klebsiella pneumoniae* [25, 26].

It is known that butyryl-CoA is generally formed from crotonyl-CoA by the NADH dependent trans-2-enoyl-CoA reductase (Ter) enzyme (Eq. 6) [27].

6 $${\text{Crotonyl-CoA }} + {\text{NADH }}\mathop \to \limits^{\text{Ter}} {\text{NAD }} + {\text{Butyryl-CoA }}$$

But recently, Buckel and Thauer [28] proposed a new indirect route of H_2_ formation in *C. pasteurianum* from NADH and ferredoxin in two steps, catalyzed successively by the ferredoxin-dependent butyryl-CoA dehydrogenase/electron transferring flavoprotein complex (BCdH-ETF) (Eq. 7) and a hydrogenase (Eq. 8).

7 $${\text{Fd}}_{\text{ox}} + 2 {\text{NADH }} + {\text{Crotonyl-CoA }}\mathop{\longrightarrow}\limits^{{{\text{BCdH}} - {\text{ETF}}}} {\text{Fd}}_{\text{red}} + 2 {\text{NAD }} + {\text{Butyryl-CoA }}$$

8 $$\text{Fd}_{\text{red}} + 2{\text{H}}^{ + } \mathop{\longrightarrow}\limits^{{{\text{H}}_{2} - {\text{ase}}}} {\text{Fd}}_{\text{ox}} + {\text{H}}_{2}$$

Since the measured H_2_ production values were significantly higher than the theoretically calculated ones based on Eqs. 7 and 8 (see Fig. 3b), it is reasonable to assume that in *C. pasteurianum* DSMZ 525, BCdH-ETF together with Ter is actively involved in the step of converting crotonyl-CoA to butyryl-CoA, giving rise to an additional source of H_2_ formation. Thus, with this new suggested butyryl-CoA formation route one mole NADH_2_ is additionally required for the formation of one mol butanol or one mol butyrate, accompanied with the formation of one mole more H_2_, in addition to the H_2_ formation counted in Eq. 5. Consequently, the calculation of reducing equivalent recovery should be modified as follows (Eq. 9), by also taking into account the difference of calculated and measured H_2_ values (c_∆H2_), representing the additionally consumed NADH_2_:

9 $${\text{NADH}}_{\text{recovery}} \,( {\text{\%}}) = \frac{{{\text{c}}_{{1,3 - {\text{PDO}}}} + {\text{c}}_{{\Delta {\text{H}}2 }} }}{{2 {\text{c}}_{\text{acetat}} + 2 {\text{c}}_{\text{butyrat}} + {\text{c}}_{\text{lactate}} + 13.2 {\text{c}}_{\text{BTM}} }}$$

Using Eq. 9, a more satisfying reducing equivalent recovery of 105% under Fe+ condition and 100% under Fe− condition was obtained, giving a strong support for the involvement of the BCdH-ETF complex. Particularly, the results from Fe+ condition are in agreement with the corrected Eq. 9, where more BuOH and hydrogen were produced, and the deviation between the calculated and measured H_2_ was higher. However, this is in contrary to what was reported for *C. acetobutylicum*. For a more effective butanol production, a lower hydrogenase activity and hydrogen production was favored in *C. acetobutylicum* [29]. To shed more light on the mechanisms underlying the effect of iron on the regulation of glycerol metabolism in *C. pasteurianum*, comparative proteomic studies were performed as described below.

### Comparative proteomic analysis of the iron effect

For proteomic analysis of the effects of iron concentration on the metabolism of *C. pasteurianum*, samples were taken from the two bioreactors in the exponential growth phase (termed as Fe+ early and Fe− early, respectively) and the stationary growth phase (Fe+ late and Fe− late, respectively) (Fig. 1). Each sample was analyzed in triplicates. After 2-DE separation of the intracellular proteins, protein spots showing statistically significant changes between Fe+ early and Fe− early, Fe+ late and Fe− late, Fe+ early and Fe+ late, as well as Fe− early and Fe− late were further identified by LC–MS/MS. Proteins which were identified as single protein present in a spot on the 2-D gels are summarized in Table 2 according to their functional categories and accession numbers, together with the information of their expression changes. The existence of more than one values of fold change for a single protein indicates that this protein appeared as multiple spots on the 2-D gels.

**Table 2:** Proteins showing significant changed expression levels, compared between iron excess (Fe+) and iron limitation (Fe−) conditions, as well as between the exponential growth phase (early) and the stationary phase (late)

Accession no.	Protein name	Cluster of orthologous groups (COG)	Conserved protein domain	Spot no.	Fold change iron-related				Fold change growth-related			
					Early phase higher at		Late phase higher at		Fe+ higher at		Fe− higher at	
					Fe−	Fe+	Fe−	Fe+	Early	Late	Early	Late
Amino acid transport and metabolism												
F502_05097	Amino peptidase 1	COG1362	Lap4	298	1.9		1.7					
F502_05412	Carbamoyl phosphate synthase large subunit	COG0458	CarB	89		1.8			1.9			
F502_07028	Cysteine synthase a	COG0031	CysK	449	1.8					2.1		
F502_17572	Glutamine synthetase type III	COG3968	GlnA3	187	1.6					1.9		
				190	1.6					1.6		
F502_18676	Threonine synthase	COG0498	ThrC	312		1.6			2.2			
Carbohydrate transport and metabolism												
F502_03412	Propanediol dehydratase small	COG4910	PduE	594	1.9		1.9					
F502_07638	Subunit flavodoxin	COG0716	FldA	594								
F502_03417	Glycerol dehydratase reactivation factor large subunit	No COG		220					2.1			
F502_03937	Glycogen synthase	COG0297	GlgA	329	2.7					2.3		
F502_06067	Enolase	COG0148	Eno	695	4.1		4.0			2.0		2.0
				696	4.8		1.8			2.5		
F502_06077	Triosephosphate isomerase	COG0149	TpiA	491				1.5		2.6		
F502_06087	Glyceraldehyde 3-phosphate dehydrogenase	COG0057	GapA	409		1.6			2.4			
F502_07098	Glycoside hydrolase	COG1543		277	2.0					1.8		
F502_12758	Dihydroxyacetone kinase	COG2376	DAK1	158		1.7		2.0	1.7		2.1	
				179		1.7		1.7	2.2		2.3	
Cell cycle control/cell division												
F502_08238	Cell division protein	COG3599	DivIVA	503					2.7		2.0	
Cell wall/membrane/envelope biogenesis												
F502_00655	Peptidoglycan-binding protein	COG1388	LysM	233	7.9					6.1	1.6	
F502_01965	Spore coat protein Frelated protein	COG5577	CotF	549	1.5		2.1					1.6
Coenzyme transport and metabolism												
F502_07578	Pyridoxal biosynthesis lyase	COG0214	PdxS	460		2.5			2.2			
Energy production and conversion												
F502_05017	NifU-related domain containing protein	COG0822	IscU	519	0.4	2.4	0.6	1.6	1.3	0.8	0.9	1.1
				520		3.7		2.9	1.9		1.5	
F502_06282	Electron transfer flavoprotein subunit alpha	COG2025	FixB	440				1.5				
F502_06287	Electron transfer flavoprotein subunit alpha/beta-like protein	COG2086	FixA	487				1.7				
F502_06447	Bifunctional acetaldehyde-CoA/alcohol dehydrogenase	COG1012	AdhE	119				2.1		3.7		1.6
				715	1.5			2.9		5.3		
				717	1.6			3.0		4.7		
F502_07493	Nitroreductase	COG0778	NfnB	537	2.3		2.9					1.8
F502_07643	Pyruvate: ferredoxin (flavodoxin) oxidoreductase. homodimeric	COG0674	PorA	70		2.4		2.4	1.5			
				75				2.4	0.6	1.8		
				76				2.7		1.9		
				77		1.5		2.3				
				87		2.0		2.8			1.5	
				90		2.2		2.8	1.6		2.0	
F502_07648	Pyruvate: ferredoxin oxidoreductase	COG0674	PorA	40				2.3		1.6		
				57				2.3		2.1		
				58		1.5		2.4		2.1		
				59		2.1		2.8				
F502_09238	Rubredoxin/flavodoxin/oxidoreductase	COG0426	NorV	131	1.7					2.0		
				730						2.0		1.6
F502_09488	Hydratase (aconitase A)	COG1048	AcnA	156		2.4		2.5	1.9		2.0	
F502_11871	Butyrate kinase	COG3426	Buk	383	1.6					1.7		
F502_11976	Pyruvate carboxylase	COG1038	PycA	71		1.5		2.7		1.7		
F502_12091	F0F1 ATP synthase subunit beta	COG0055	AtpD	645	2.1		1.9					
				646	2.2		2.4					
F502_12878	Desulfo ferrodoxin	COG2033	SorL	611		2.2		2.8				
F502_13493	Flavodoxin	COG0716	FldA	575	14.3		8.0			1.6		
F502_14390	[Fe] hydrogenase	COG4624	Nar1 PurB	303		2.1			2.5			
F502_04707	Adenylosuccinate lyase	COG0015		303								
F502_15080	Rubrerythrin	COG1592	YotD	558		2.2		4.5		2.0		
F502_16610	Glycolate oxidase	COG0277	GlcD	315				1.8		2.3		1.9
F502_18287	Hydrogenase-1	COG1034	NuoG	223		4.5		1.6	2.3			
				224		5.1		1.9	3.6			
F502_18651	NADP-dependent glyceraldehyde-3-phosphate dehydrogenase	COG1012	AdhE	736	2.7		1.5			2.1		
F502_19556	Formate acetyltransferase	COG1882	PflD	174	4.5		5.7					
				175	8.0		6.5					
				178	2.5						3.4	
				181	3.7				1.7		7.4	
Function unknown/general function prediction only												
F502_02435	Aldo/keto reductase	COG1453		362		2.6			2.9			
F502_05012	Hypothetical protein (GGGtGRT protein)	No COG		400		7.1		2.2	3.0			
F502_05962	Hypothetical protein	No COG		635	2.0					1.5		
F502_06682	Hypothetical protein	COG2607		318		3.4		3.8	1.7		2.0	
F502_15420	Hypothetical protein	No COG		597	1.7		2.8		1.5			
F502_16320	Hypothetical protein	COG0393	YbjQ	637	2.3		2.0					
Lipid transport and metabolism												
F502_06297	3-Hydroxybutyryl-CoA dehydratase	COG1024	CaiD	472		2.1			2.2			
F502_10483	Biotin carboxylase	COG0439	AccC	302	2.5			1.6		2.1	1.9	
Nucleotide transport and metabolism												
F502_17300	Bifunctional phosphoribosylaminoimidazolecarboxamide formyltransferase/IMP cyclohydrolase	COG0138	PurH	281					1.7		1.6	
Posttranslational modification/Protein turnover/Chaperones												
F502_03242	Heat shock protein (molecular chaperone GrpE)	COG0576	GrpE	481				2.1		2.2		
F502_03247	Molecular chaperone DnaK	COG0443	Dank	217	1.6					1.8		
F502_03987	peptidase	COG1026	Cym1	106	2.3					2.1		
				107	3.6		1.6			2.4		
F502_05557	ATP-dependent Clp protease ATP-binding subunit	COG0542	ClpA	165	1.8		2.1					
				168			1.9		1.7			
F502_06242	Chaperonin	COG0459	GroEL	258	1.6					2.0		
F502_06247	Co-chaperonin	COG0234	GroES	613				1.5		2.6		
F502_07608	Thij/PfpI family protein	COG0693	ThiJ	553	1.8		1.6					
F502_10228	Heat shock protein (molecular chaperone IbpA)	COG0071	IbpA	600			2.5			3.0		6.9
F502_15425	Heat shock protein 90	COG0326	HtpG	196	2.2					2.4		
				206	1.6					1.5		
F502_18446	Clpb protein	COG0542	ClpA	138	1.8			1.5		2.8		
				716	2.5			2.4		4.4		
F502_18743	ATPase with chaperone activity clpC. two ATP-binding domain protein	COG0542	ClpA	147	2.1		2.1			2.0		2.0
			ClpA	149	2.5		1.7			2.4		1.6
Signal transduction/stress response/defense mechanism												
F502_04082	GTP-binding protein	COG1217	TypA	47					1.7		1.8	
				148		1.7			2.4		2.1	
F502_07703	Chemotaxis histidine kinase. CheA (contains CheW-like adaptor domain)	COG0643	CheA	155	3.6		2.2				1.6	
F502_10768	Lipid hydroperoxide peroxidase	COG2077	Tpx	572	3.9		2.2			2.5		
F502_13258	CBS domain-containing protein	COG0517	CBS	606	4.5		4.8			1.7		1.9
F502_14770	Serine protein kinase	COG2766	PrkA	193	4.7		1.5			3.4		
				197	3.7		2.6			2.1		
				200	6.2		1.9			3.6		
				201	3.8					2.6		
F502_16565	Nitrogen regulatory protein P-II	COG0347	GlnK	638	3.1		1.8					
F502_17612	Alkyl hydroperoxide reductase	COG0450	AhpC	567	5.3		2.9			2.4		
F502_17637	Spore coat protein	COG3546	CotJC	548	2.3		3.9					2.1
F502_18092	Stage V sporulation protein T	COG2002	AbrB	544	6.1		4.5			1.7		
Transcription/Defense mechanisms												
F502_12326	Transcription accessory protein	COG2183	Tex	160		1.7	1.8		4.7		1.5	
Translation/Ribosomal structure and biogenesis												
F502_04537	30S ribosomal protein S2	COG0052	RpsB	497		2.1		2.1	2.7		2.7	
				500		2.4		1.9				
F502_06817	Ribosomal 5S rRNA E-loop binding protein Ctc/L25/TL5	COG1825	RplY	504	3.5					3.9		
F502_12196	Ribosome-associated protein Y (PSrp-1)	COG1544	RaiA	565	2.4					2.4		
F502_18808	Elongation factor Tu	COG0050	TufB	327		1.7		1.5	1.5		1.4	
F502_18833	50S ribosomal protein L1	COG0081	RplA	681		2.4		1.5	2.0			
F502_18948	50S ribosomal protein L5	COG0094	RplE	752		2.1			1.8			
F502_18963	50S ribosomal protein L6	COG0097	RplF	753		2.4			2.3			

*^1^ COG: according to the annotation for *C. pasteurianum* DSMZ 525 by BioCyc database collection (http://www.biocyc.org/organism-summary?object=CPAS1262449)

*^2^ Conserved Protein Domain Family: according to the definition by NCBI Conserved Domains and Protein Classification (http://www.ncbi.nlm.nih.gov/Structure/cdd/cdd.shtml)

### The pyruvate acetyl-CoA node: a focal point in the metabolism of *C. pasteurianum*

**Fig. 4:**
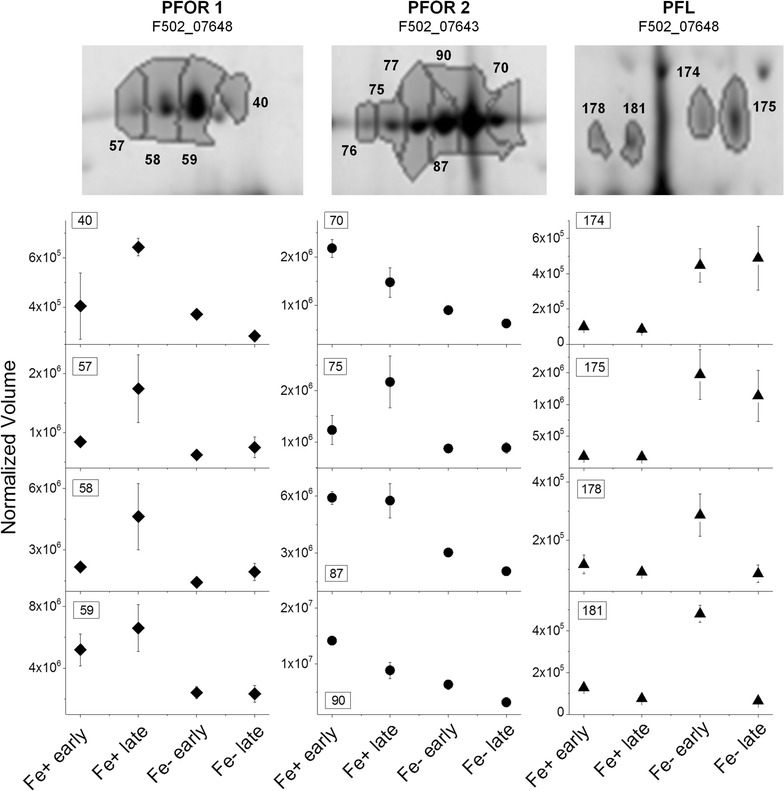
Expression patterns of enzymes catalyzing the conversion of pyruvate to acetyl-CoA under Fe+ and Fe− conditions at exponential growth phase (early) and stationary phase (late). PFOR 1 (F502_07648, pyruvate: ferredoxin oxidoreductase) and PFOR 2 [F502_07643, pyruvate:ferredoxin (flavodoxin) oxidoreductase] are up-regulated at Fe+ ; PFL (pyruvate formate lyase) is up-regulated at Fe−

The conversion of pyruvate to acetyl-CoA linking glycolysis to TCA cycle is a fundamental metabolic step of living organisms in general. In anaerobes, pyruvate can be metabolized through a variety of pathways but it is often oxidized to CO_2_ and acetyl-CoA with the concomitant reduction of a low-potential redox protein, like ferredoxin or flavodoxin. The enzyme responsible for this oxidative decarboxylation of pyruvate in many anaerobic bacteria and archaea is pyruvate: ferredoxin oxidoreductase (PFOR). PFORs contain thiamin pyrophosphate (TPP) for the cleavage of carbon–carbon bonds next to a carbonyl group, as well as iron-sulfur clusters for electron transfer (see [30, 31] and references therein). For example, PFOR from *C. pasteurianum* W5 (ATCC 6013) was characterized to be an air-sensitive homodimer with each subunit containing eight iron atoms in two [4Fe–4S] clusters, for which pyruvate is the best substrate found among several α-ketoacids [30]. In the genome of *C. pasteurianum* DSMZ 525, three homologue enzymes of PFOR are present, namely two pyruvate ferredoxin oxidoreductases (F502_01955 and F502_07648), and one pyruvate:ferredoxin (flavodoxin) oxidoreductase (F502_07643) [32]. In this study, F502_07648 (termed as PFOR1) and F502_07643 (termed as PFOR2) were identified among the proteins showing significant expression changes. On the 2-D gels both PFORs appeared as a chain of spots (Fig. 4). Protein identification showed that the spots 70, 75, 76, 77, 87, 90 are pI isoforms of the homodimeric protein pyruvate: ferredoxin (flavodoxin) oxidoreductase (F502_07643), whereas the spots 40, 57, 58 and 59 are pI isoforms of pyruvate:ferredoxin oxidoreductase (F502_07648). F502_07643 and F502_07648 are homologous proteins with a sequence identity match of 66% and positive match of 81%. They have nearly identical molecular weights but the pI value of F502_07648 is more basic than that of F502_07643, which was also obvious on the 2-D gels. Although previous studies showed that under conditions of iron limitation many anaerobes synthesize flavodoxins as substitution of ferredoxins for many enzymatic reactions [28], all the isoforms of the two PFORs showed, in general, higher expression at iron excess than at iron limitation (Fig. 4). The expression patterns of the isoforms of PFOR1 were similar to each other, with the highest expression at Fe+ late. In contrast, the expression patterns of the isoforms of pyruvate:ferredoxin (flavodoxin) oxidoreductase (PFOR2) were different to each other. While the expression level of the spot 87 in the middle of the spot chain did not change between Fe+ early and Fe+ late, the more basic isoforms (spots 70 and 90) showed higher expression at Fe+ early, and the more acidic isoforms (spots 75, 76 and 77) were up-regulated at Fe+ late which was similar to the expression pattern of PFOR1. Therefore, based on the proteomic results alone, it is not clear whether these two PFORs function in synchronization or are differently regulated in response to iron availability. Furthermore, whether PFOR2 transfers the electrons generated during the decarboxylation reaction to a ferredoxin or flavodoxin remains elusive.

Beside the ferredoxin (flavodoxin)-dependent PFORs, acetyl-CoA can be synthesized from pyruvate through the pyruvate formate-lyase (PFL) with the formation of formate. There are three genes (F502_19556, F502_15690, F502_15710) in the genome of *C. pasteurianum* DSMZ 525 being annotated to encoding enzymes functioning as pyruvate formate lyase (PFL). Only one of these PFLs (F502_19556, also named formate acetyltransferase) was unambiguously identified in four protein spots (Fig. 4). Interestingly, the molecular weight of the two acidic isoforms (spots 178 and 181) appeared lower than that of the two basic isoforms (spots 174 and 175). Compared to the iron excess condition, where the expression of PFL was nearly not detectable, all these four PFL isoforms were significantly but differently up-regulated under the iron limitation condition. While the two acidic isoforms showed 2.5 and 3.7 folds increased abundances only in the Fe− early sample, the expression of the two basic isoforms were up to eightfold strongly up-regulated in the Fe− early sample and about sixfold in Fe− late sample. Thus, under iron limitation, it was apparently favorable for *C. pasteurianum* to use the PFL-catalyzed reaction for the conversion of pyruvate to acetyl-CoA. Correspondingly, under this condition the formate yield was clearly higher than that under the Fe+ condition, namely 0.25 ± 0.05 g/g biomass in contrast to 0.08 ± 0.02 g/g biomass. Nevertheless, the expression levels of the two PFORs, especially the pyruvate:ferredoxin (flavodoxin) oxidoreductase (PFOR2), were visibly higher than that of PFL. Since protein synthesis is an energy-demanding process, cells usually do not produce useless enzymes in noticeable amounts. The presence of the two PFORs under Fe− condition may point to a fact that, in the absence of iron, the two PFORs, especially the pyruvate: ferredoxin (flavodoxin) oxidoreductase (PFOR2), probably use flavodoxin instead of ferredoxin as the electron acceptor. Indeed, the expression of a flavodoxin (F502_13493) was strongly up-regulated under iron limitation for 14.3 folds in the exponential growth phase and remained high even after entering the stationary phase (8.0 fold higher in Fe− late than in Fe+ late). However, whether or not the up-regulated expression of this flavodoxin was coupled to the functionality of the PFORs remains to be verified. In case it is, it did not help much in sustaining the production of butanol under the Fe− condition.

### Regulation of the ferredoxin pool

For the proper function of PFORs, ferredoxin_(red)_, which is generated in the PFOR-catalyzed pyruvate oxidative decarboxylation reaction, must be oxidized to regenerate ferredoxin_(ox)_. *C. pasteurianum* DSMZ 525 possesses a big number of ferredoxins and the regeneration of ferredoxin_(ox)_ can be achieved using different electron acceptors. The fact that the redox potentials of ferredoxins (−400 mV) are in the range of H_2_ electrodes (−414 mV, at pH 7) reveals that in most energy metabolisms where ferredoxins are active, H_2_ is also involved, either as substrate or as product. In general, nitrogenases and hydrogenases are the two enzyme classes capable of hydrogen production in Clostridia [33]. But Hallenbeck and Benemann [34] reported that hydrogenases are much more efficient, with more than 1000 times higher turnover than nitrogenases. Hydrogenases are divided into two main groups in *Clostridia* based on their metaollocenter composition, namely [NiFe] and [FeFe] hydrogenases [33]. In this study, the expression of hydrogenase-1 (F502_18287), which belongs to the [FeFe] group, was highly up-regulated under iron excess, showing up to fivefolds higher expression level in the exponential growth phase under the Fe+ condition compared to the Fe− condition. After entering the stationary phase the expression of hydrogenase-1 (H_2_-ase) was down-regulated for two to threefolds under the Fe+ condition, which could be possibly in response to a depletion of the intracellular iron pool in the Fe+ late sample required for this [FeFe]-hydrogenase. However, it was still nearly twofolds higher than its expression level under the Fe− condition. An additional [FeFe] hydrogenase (F502_14390) was also identified which showed expression regulations similar to that of the hydrogenase-1 (F502_18287). Nevertheless this hydrogenase did not appear as a spot containing only a single protein on the 2-D gels and therefore could not be quantified for comparison. The higher expression of hydrogenase-1 (F502_18287) coincided with the higher H_2_ production in the fermentation culture under iron excess and should have significantly contributed to the regeneration of ferredoxin_(red)_ to ferredoxin_(ox)_.

However, it is to notice that the regulation of hydrogenase-1 (F502_18287) expression is rather in agreement with that of the basic iso-forms of the ferredoxin (flavodoxin)-dependent PFOR2 than with the expression patterns of the ferredoxin-dependent PFOR1. Therefore, it is tempting to suggest that under the given experimental conditions the hydrogenase-1 catalyzed reaction should not be the only route of ferredoxin_(ox)_ regeneration. The PFORs-catalyzed pyruvate oxidation to acetyl-CoA might be coupled with other but yet unknown ferredoxin_(ox)_ regenerating reaction(s) catalyzed either by other unidentified hydrogenases (at least 5 genes in the genome of *C. pasteurianum* DSMZ 525 encode hydrogenases) or ferredoxin reductases. In addition, it has also been reported that PFOR can transfer the electrons generated in the decarboxylation reaction directly to protons to generate molecular hydrogen [35].

**Fig. 5:**
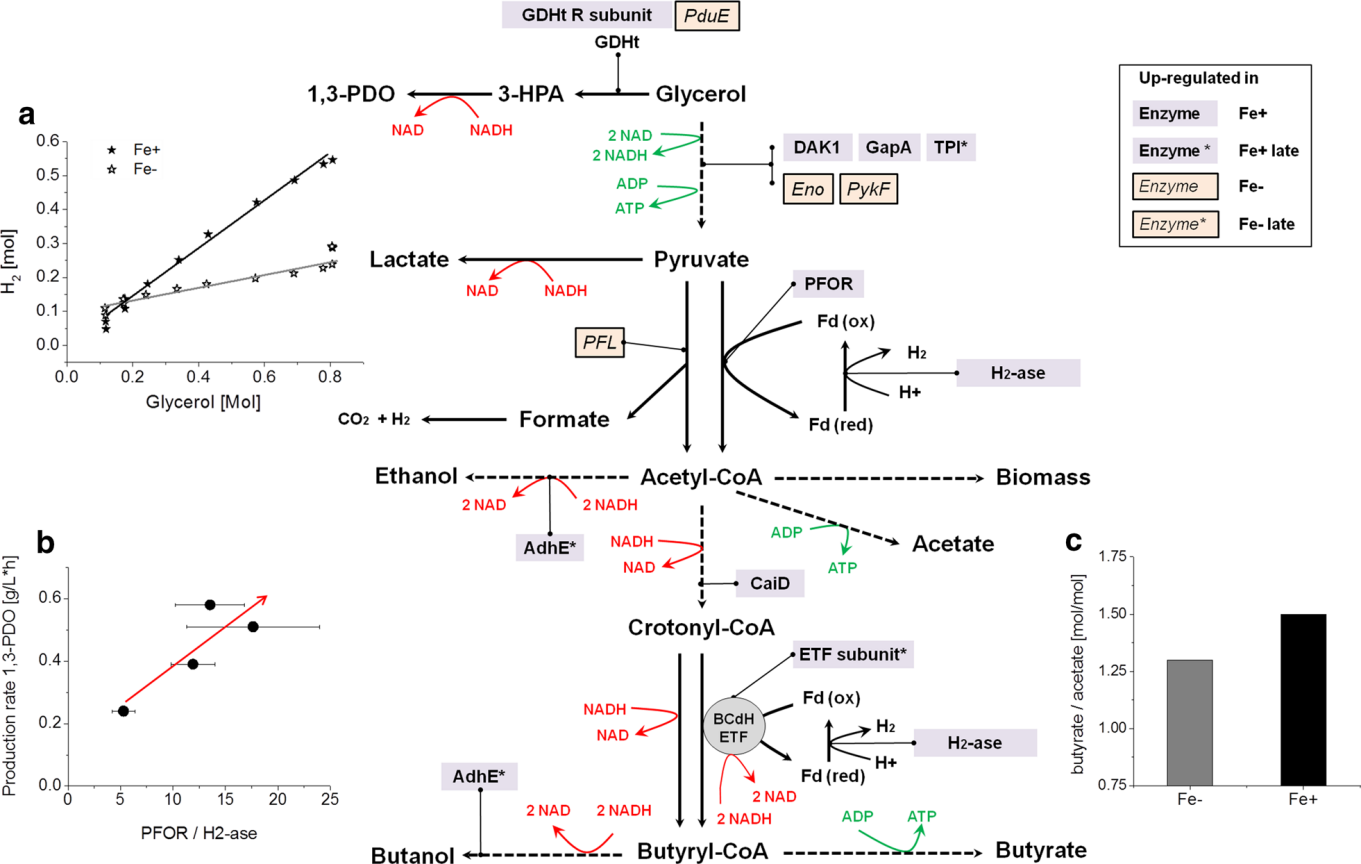
Revised metabolic pathway of glycerol bioconversion to 1,3-propanediol and n-butanol in *C. pasteurianum.* **a** Molar formation of H_2_ over consumed glycerol **b** 1,3-PDO productivity over the ratio PFOR/H_2_-ase **c** Molar ratio of butyrate to acetate under Fe+ and Fe− conditions

Within the cells of anaerobes including Clostridia, 90% of ferredoxins were reported to be present in reduced form, allowing them to serve as electron donors in different reactions [28]. In general, this is achieved in *C. pasteurianum* by the following three ferredoxin-dependent redox reactions: the oxidation of pyruvate to acetyl-CoA and CO_2_ (−500 mV), the oxidation of formate to CO_2_ (−430 mV, [36]) and the flavoprotein based electron bifurcation involved in the reduction of crotonyl-CoA to butyryl-CoA (Eq. 7). On the other hand, the oxidation of Fd_red_ by NAD is excluded due to the absence of the ferredoxin: NAD oxidoreductase activity [28]. Therefore, hydrogen production via hydrogenase should be a main route of Fd_ox_ regeneration in *C. pasteurianum* DSMZ 525. Based on this assumption, we compared the hydrogen yield from glycerol between the Fe− and the Fe+ conditions. As shown in Fig. 5a, hydrogen yield decreased significantly from 0.75 mol/mol glycerol under Fe+ condition to 0.21 under Fe− condition, which was in agreement with the higher expression of hydrogenase-1 under the Fe+ condition.

In addition, as described in the above redox balance analysis, a possible involvement of a ferredoxin-dependent butyryl-CoA dehydrogenase/electron transferring flavoprotein complex (BCdH-ETF) in H_2_ production was proposed. In the BCdH-ETF catalyzed reaction electron transfer flavoproteins (ETFs) are involved in the reduction of crotonyl-CoA to butyryl-CoA, coupled with ferredoxin_(ox)_ reduction by bifurcating electrons from NADH (Fig. 5) [28, 37]. In this proteomic study, two ETFs, namely ETFs subunit alpha (F502_06282) and subunit alpha/beta-like protein (F502_06287), were identified among the most abundant proteins regardless of the iron availability; however, their abundances were slightly higher (1.5–1.7 folds) in the late phase of the Fe+ culture compared to the Fe− late condition. This might indicate a relative increase in the oxidized ferredoxin pool necessary to carry out the BCdH-ETF reaction and also contributed to the stronger H_2_ production in the late fermentation phase under Fe+ condition.

Nevertheless, not only the hydrogenase-1 but also the two PFORs were down-regulated under Fe− condition compared to that under Fe+ condition. Therefore, the relative changes of the expression levels of the two enzymes might be indicative of the overall Fd_ox_ regeneration state (Fig. 5). The expression levels shown as the protein spot intensities of both enzymes under Fe− and Fe+ conditions at the two time points were thus compared. As shown in Fig. 5b, the relative expression of PFOR to hydrogenase-1 (H_2_-ase) showed a positive correlation with the 1,3-PDO production rate. The decrease in the Fd_ox_ fraction under Fe− condition due to reduced H_2_-ase presence will decrease the Fd_ox_ coupled synthesis of butyryl-CoA catalyzed by the BCdH-ETF complex, a crucial step in butyrate and especially butanol biosynthesis. Moreover, the intermediate acetyl-CoA will be favorably channeled into the Fd_ox_-independent acetate formation route than the Fd_ox_-dependent butyrate formation route, as shown by the increase in the butyrate/acetate ratio from 1.3 at Fe− to 1.5 at Fe+ (Fig. 5c). Consequently, it seems that under Fe− condition, Fd_ox_ dependent conversion steps are reduced and the resulting free reducing power, usually needed for butanol formation, could be redirected for the sake of redox balance to the production of 1,3-PDO and lactate. Indeed, the overall yield of 1,3-PDO and lactate were much higher under iron limitation than under iron excess (Table 1). Nevertheless, lactate dehydrogenase catalyzing the conversion of pyruvate to lactate or 1,3-propanediol dehydrogenase catalyzing the formation of 1,3-propanediol from 3-HPA were not found among the proteins showing significant changes in expression level. Both enzymes are not involved in energy production but constitute the cell’s back-up for stabilizing an internal redox balance, and hence their constitutive production may be a mechanism to withstand sudden perturbations in the NADH/NAD ratio. Nevertheless, it should also bear in mind that higher or lower protein level does not always means higher or lower enzyme activity.

### Glycerol conversion to 1,3-propanediol

**Fig. 6:**
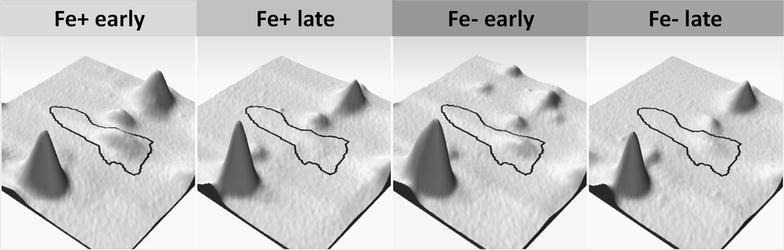
Expression pattern of the large subunit of glycerol dehydratase reactivating factor (GDHtR) under Fe+ and Fe− conditions as well as early and late sampling point

In general the bioconversion of glycerol to 1,3-PDO takes place in two steps, catalyzed successively by glycerol dehydratase (GDHt) and 1,3-propanediol dehydrogenase (PDOR) (Fig. 5). It is known that glycerol dehydratase is the rate-limiting enzyme in this bioconversion. All the three subunit of GDHt encoded by *pduC* (F502_03402), *pduD* (F502_03407) and *pduE* (F502_03412) were identified but unfortunately not as single protein spots and, therefore, could not be quantified. Instead, the large subunit of glycerol dehydratase reactivating factor (GDHt reactivase, GDHtR) was identified in the spot containing this single protein (Fig. 6). GDHtR is a molecular chaperone participating in the reactivation of inactivated GDHt in the presence of ATP and Mg^2+^ [38, 39]. The expression pattern of GDHtR indicates rather a correlation of GDHtR expression to cell growth phase than to iron availability. Among the four samples compared by proteomics, the highest expression level of GDHtR was present in the iron excess culture in the middle exponential growth phase (Fe+ early) showing the highest specific growth rate (µ = 0.22). At this sampling time point, the culture under iron limitation (Fe− early) already entered late exponential growth phase with reduced specific growth rate (µ = 0.07), accompanied with lower GDHtR level. The GDHtR abundance was further reduced to merely detectable levels in the stationary phase (Fe+ late and Fe− late), where the production of 1,3-PDO stagnated. 1,3-propanediol dehydrogenase (PDOR), the responsible enzyme for the conversion of 3-HPA to 1,3-PDO, was one of the highly abundant proteins on the 2-D gels and did not show significant expression changes under the different conditions (data not shown).

## Conclusion

The iron content in the fermentation medium was shown to influence the product formation, especially the 1,3-propanediol and butanol distribution, in *Clostridium pasteurianum* DSMZ 525 grown on glycerol. Compared to the fermentation under iron limitation, it was shown that the butanol to 1,3-propanediol ratio increased almost fivefold in fermentation under iron excess. To better understand the effect of iron on the regulation of the cell metabolism, physiological and proteomic analyses were performed. Several enzymes like pyruvate: ferredoxin oxidoreductase (PFOR), hydrogenase and bifunctional acetaldehyde-CoA/alcohol dehydrogenase among others were found to be up-regulated under iron excess conditions. The differential expression of PFORs and a pyruvate formate lyase (PFL) in response to iron availability highlighted the impact of iron on the crucial step of the central carbon metabolism in *C. pasteurianum*. The importance of a hydrogenase in the regeneration of oxidized ferredoxin and therefore the maintaining of the redox balance was confirmed by its strong up-regulation under the iron excess condition. Beside the release of molecular hydrogen in the pyruvate to acetyl-CoA step catalyzed by PFORs, stoichiometric analysis showed a possible H_2_ production coupled to the reaction catalyzed by the ferredoxin-dependent butyryl-CoA dehydrogenase/electron transfer flavoprotein complex (BCdH-ETF). Indeed, proteomic analysis revealed the up-regulation of two electron transfer flavoproteins which may be involved in this metabolic conversion step. Since both 1,3-propanediol and butanol can be used as sink for NADH, we suggest that the ratio of oxidized ferredoxin to reduced ferredoxin in addition to the NADH availability contributes to the selectivity of the products.
